# Supplementary material for: Zhamanshin astrobleme provides evidence for carbonaceous chondrite and post-impact exchange between ejecta and Earth’s atmosphere
Source: Nat Commun. 2017 Aug 9;8:227. doi: 10.1038/s41467-017-00192-5 (PMC5550458; doi:10.1038/s41467-017-00192-5)
Supplement: Supplementary file 1 — Supplementary Information [file 41467_2017_192_MOESM1_ESM.pdf]

File name: Supplementary Information
Description: Supplementary Note and Supplementary References

File name: Peer Review File
Description:

Supplementary information

Supplementary Note 1

The Zhamanshin Impact Structure, Kazakhstan (48°24'N/60°58'E) has an estimated diameter of ~13–14 km, based on remote sensing[1,2], and evaluation of earlier geophysical surveys[3-6]. This diameter, however, was attained by a gravity induced slope collapse and landslides of the original structure ~5.5–6 km in diameter in loosely consolidated sediments[7]. Rather controversial fission-track, K–Ar and Ar–Ar data indicate an impact event at 1.0 ± 0.15 Ma[8-11]. Target lithologies in Zhamanshin belong to two main units: (i) ~150 m thick platform cover (Cretaceous sandstones and marls, covered by Paleogene sands, clays and locally sandstones), and (ii) basement, consisting of two folded and faulted series, lower Neoproterozoic to Lower Paleozoic volcanosedimentary series and overlying Upper Paleozoic volcanosedimentary series with conglomerates, sandstones and andesitic–basaltic volcanic rocks. Details on general geology of the area, sample nomenclature and description are summarized elsewhere[7,12].

SUPPLEMENTARY REFERENCES

1 Garvin, J. B., Schnetzler, C. C. & Grieve, R. A. Characteristics of large terrestrial impact structures as revealed by remote sensing studies. *Tectonophysics* **216**, 45-62 (1992).

2 Garvin, J. B. & Schnetzler, C. C. in *Large meteorite impacts and planetary evolution.* B.O. Dressler, R.A.F. Grieve, & V.L. Sharpton, Eds., Geol. Soc. Am. Spec. Publ. **293**, 249-257 (1994).

3 Florenskii, P. V. & Dabizha, A. I. *Meteoritnyi krater Zhamanshin (In Russian).* Nauka. Moscow, Russia. 128 pp. (1980).

4 Masaitis, V. L., Boiko, Y. I. & Izokh, E. P. Zhamanshin Impact Crater (Western Kazakhstan): Additional geological data. *Lunar Planet. Sci.* **XV**, 515-516 (1984).

5 Izokh, E. P. in *Kosmicheskoe veshchestvo i Zemlya.* Yu.A. Dolgov, Ed., pp. 159-203 (Nauka, Moscow, Russia, 1986).

6 Izokh, E. P. Impaktnyi krater Zhamanshin i problema tektitov. *Geologiya i Geofyzika* **4**, 3-15 (1991).

7 Jonášová, Š. *et al.* Geochemistry of impact glasses and target rocks from the Zhamanshin Impact Structure, Kazakhstan: Implications for mixing of target and impactor matter. *Geochim. Cosmochim. Acta* **190**, 239-264 (2016).

8 Kolesnikov, E. M., Smolyar, M. I., Lebedeva, L. M., Shykolyukov, Y. A. & Izokh, E. P. in *2nd Int. Conf. Nat. Glasses.* J. Konta, Ed., pp. 203-205 (Charles University, Prague, Czech Republic, 1988).

9 Deino, A. L., Becker, T. A. & Garvin, J. B. Laser-fusion $^{40}Ar/^{39}Ar$ ages of acid zhamanshinite. *Lunar Planet. Sci. Conf.* **XXI**, 271-272 (1990).

10 Koeberl, C. & Storzer, D. in *2nd Conf. Nat. Glasses.* J. Konta, Ed., pp. 207-213 (Charles University, Prague, Czech Republic, 1988).

11 Storzer, D. & Koeberl, C. Fission track evidence for multiple source components of Zhamanshin impactites, and new fission track ages. *Meteoritics* **24**, 328-329 (1989).

12 Mizera, J., Řanda, Z. & Tomandl, I. Geochemical characterization of impact glasses from the Zhamanshin crater by various modes of activation analysis. Remarks on genesis of irghizites. *J. Radioanal. Nucl. Chem.* **293**, 359-376 (2012).
